# Machine learning driven multi-omics analysis of the genetic mechanisms behind the double-coat fleece formation in Hetian sheep

**DOI:** 10.3389/fgene.2025.1582244

**Published:** 2025-06-11

**Authors:** Yanwei Zhang, Wenrong Li, Xinming Xu, Mengwan Xie, Liping Tang, Peiyu Zheng, Nannan Song, Lijuan Yu, Jiang Di

**Affiliations:** ^1^ Key Laboratory of Evaluation and Utilization of Livestock and Poultry Resources (Sheep) of the Ministry of Agriculture and Rural Areas, Institute of Animal Sciences, Xinjiang Academy of Animal Sciences, Xinjiang, China; ^2^ Key Laboratory of Animal Biotechnology of Xinjiang, Institute of Biotechnology, Xinjiang Academy of Animal Sciences, Xinjiang, China

**Keywords:** Hetian sheep, Chinese merino sheep, coat fleece type, multi-omics, machine learning

## Abstract

**Introduction:**

The double-coated fleece is crucial for the adaptability and economic value of Hetian sheep, yet its underlying molecular mechanisms remain largely unexplored.

**Methods:**

We integrated genome and transcriptome data from double-coated Hetian sheep and single-coated Chinese Merino sheep. Candidate genes associated with coat fleece type and environmental adaptation were identified using combined selective sweep and differential expression analyses. Subsequent analyses included Gene Ontology (GO) and Kyoto Encyclopedia of Genes and Genomes (KEGG) pathway enrichment, protein-protein interaction (PPI) network construction, and machine learning-based screening.

**Results:**

Selective sweep and differential expression analyses identified 101 and 106 candidate genes in Hetian sheep and Chinese Merino sheep, respectively. Enrichment analyses revealed these genes were primarily involved in pathways related to wool growth and energy metabolism. PPI network analysis and machine learning identified IRF2BP2 and EGFR as key functional genes associated with coat fleece type.

**Discussion:**

This study enhances understanding of the genetic mechanisms governing double-coated fleece formation in Hetian sheep. The identification of key genes (IRF2BP2, EGFR) and the methodological approach provide valuable insights for developing machine learning-driven multi-omics selection models in sheep breeding.

## 1 Introduction

Hetian sheep is a unique local breed from Hetian, Xinjiang, China, known for its high-quality carpet wool. Hetian carpets, made from Hetian sheep wool, are famous worldwide. The breed was listed in the National List for the Protection of Livestock and Poultry Genetic Resources in 2006. Hetian sheep are adapted to the temperate continental desert climate, characterized by drought resistance, heat tolerance, ability to thrive on coarse feed, strong disease resistance, and exceptional adaptability to harsh environments (Han et al., 2023). One of the most distinctive features of Hetian sheep is their double-coated fleece, which includes coarse wool, fine wool, and heterotypical hair. These fibers differ in structure, with heterotypical hair being particularly notable for its interrupted medullary layers, a characteristic that contributes to the plush and durable texture of Hetian carpets (Chen et al., 2024; Shi et al., 2022a). This complex fleece structure raises important questions about the genetic mechanisms behind its formation.

Despite the recognized significance of Hetian sheep’s unique fleece, research on its double-coated fleece and heterotypical hair remains limited, primarily due to the complexity of the underlying genetic mechanisms (Wang et al., 2025; Shi et al., 2022b; Wang et al., 2021). Multiple genes interact in the development of double-coated fleece, and environmental factors, such as temperature variations and drought conditions, further complicate the genetic analysis. These challenges highlight the need for more focused research into the genetic basis of Hetian wool.

In recent years, multi-omic analysis methods have been increasingly applied in sheep breeding (Wang et al., 2023; Li et al., 2024; Xu et al., 2023). By integrating data across various omics levels, researchers can gain a more comprehensive understanding of the genetic and biological mechanisms underlying target traits, enhancing the precision and efficiency of breeding programs. For example, Banos et al. (2017) integrated genomic and transcriptomic data to identify 14 candidate genes related to innate immunity in Chios sheep. Zhao et al. (2021) combined transcriptomic and methylation datasets from Merino sheep skin to reveal differential expression profiles across four genotypes at six hair follicle developmental stages. They identified key transcripts involved in hair follicle development through regulatory network and gene co-expression analyses, and predicted that transcription factors (e.g., *KLF4*, *LEF1*, *HOXC13*, *RBPJ*, *VDR*, *RARA*, and *STAT3*) play stage-specific roles in hair follicle morphogenesis. In another study, Wang et al. (2020) demonstrated that the expression of certain hair follicle differentiation genes and transcription factors (TFs) in sheep was negatively correlated with DNA methylation levels, using integrated RNA-seq and WGBS analysis. These findings suggest that these genes and TFs may regulate hair morphogenesis by influencing the expression of related genes.

Machine learning has also gained traction in genetic breeding, particularly in genome-wide selection, gene network analysis, and multi-omics data interpretation (Tırınk et al., 2023; Hamadani et al., 2022; Shahinfar et al., 2019). For instance, Kirchner et al. (2004) employed the decision tree (DT) algorithm to classify and predict highly reproductive sows based on characteristics such as total litter size, live litter size, and healthy litter size, achieving promising prediction results. Piles et al. (2019) applied machine learning methods to molecular-level transcriptomic data to deeply explore candidate genes influencing pig feeding efficiency. Guo et al. (2024) utilized interpretable machine learning models alongside comparative transcriptomics to identify unique factors in sheep strongly associated with muscle growth. Additionally, Farhadi et al. (2023) investigated differences in fat deposition between fat-tailed and fine-tailed sheep breeds, using meta-analysis and machine learning techniques to identify three specific genes (*POSTN*, *K35*, and *SETD4*).

In this study, we employed a combination of multi-omics and machine learning techniques to investigate the genetic mechanisms underlying the formation of double-coated fleece. Our goal was to provide a scientific basis for precision breeding. Specifically, we selected Chinese Merino sheep (characterized by single-coated and homogeneous fleece) as a control and conducted selective sweep and differential expression analyses by integrating genomic and transcriptomic data. Additionally, we applied machine learning models, such as Neural Networks (NN), to analyze these multidimensional datasets. The aim of this approach was to uncover the regulatory networks associated with double-coated fleece traits in Hetian sheep, identify key functional genes, and explore the complex relationships between these traits and environmental adaptability.

## 2 Materials and methods

### 2.1 Ethics statement

All experimental protocols in this study (including the sample collection protocol), were approved by the Ethics Committee of Institute of Animal Husbandry, Xinjiang Academy of Animal Husbandry Sciences (China) under permission no. 2025001.

### 2.2 Animals and whole genome sequencing of pools

**FIGURE 1:**
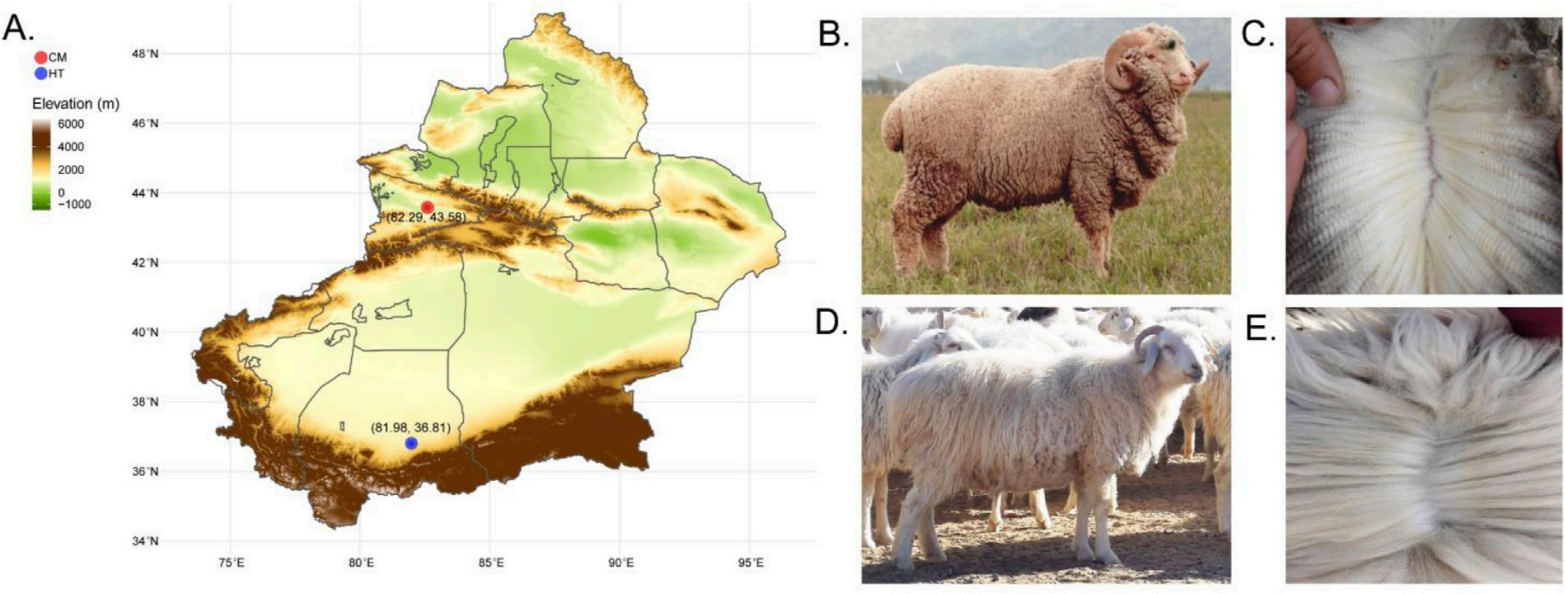
Geographical distribution of Hetian and Chinese Merino sheep, sampling locations and coat characteristics. **(A)** Geographic distribution and sampling locations. **(B)** Chinese merino sheep. **(C)** Single-coated fleece from Chinese Merino sheep. **(D)** Hetian sheep. **(E)** Double-coated fleece from Hetian sheep.

A total of 49 healthy two-year-old ewes of different coat fleece types were included in the study. Of these, 24 Hetian sheep (double-coated fleece, HT group) were from Tumuya village, Oytograk township, Yutian county, Xinjiang, and 25 Chinese Merino sheep (single-coated fleece, CM group) were from the Gongnaisi breeding sheep farm, Xinjiang (Figure 1).

Genomic DNA was extracted from the ear tissue of each sheep using the QIAamp DNA Mini Kit (QIAGEN, 51304, Hilden, Germany). DNA from each group member was pooled in equimolar amounts (2 μg/sample) to construct the two pair-end sequencing libraries (insert sizes approximately ∼0.5 kb, with effective insert sequencing concentration >2 nM). Library construction and sequencing were performed on an Illumina HiSeq 2000TM platform supplied by Beijing Novogene Bioinformatics Technology Co.

### 2.3 Genome sequence processing, mapping and SNP analysis

Raw paired reads were preprocessed to remove adapters and low-quality sequences based on the following criteria: (a) reads with more than 50% of bases having a Qphred score ≤ 5; (b) reads containing ≥10% unidentified nucleotides (N); and (c) reads with >10 nucleotides aligning to adapter sequences. High-quality reads were then mapped to the sheep reference genome (ARS-UI_Ramb_v2.0) using BWA, and the resulting SAM files were cleaned with SAMtools (v1.21) (Danecek et al., 2021) to remove duplicates. Variants were further identified using GATK (v4.1.9.0) (McKenna et al., 2010) with HaplotypeCaller, CombineGVFs, and GenotypeGVFs. To annotate SNPs and classify mutations (nonsense, nonsynonymous, and synonymous), ANNOVAR (Wang et al., 2010) was employed. Additionally, loci were refined using VCFtools (Danecek et al., 2011) to exclude those with a minor allele frequency (MAF) < 5% or a genotype deletion rate >5%

### 2.4 Selective sweep analysis

Selective sweep analysis was conducted using CM as the reference and HT as the target population to identify genes associated with wool traits. To screen genomic regions, total SNP counts were calculated for different window sizes (10, 20, 30, 40, and 50 KB). When the window size exceeded 40 KB, the SNP count dropped below 20 (Supplementary Figure S1), indicating that a 40 KB window was optimal for the analysis.

The differentiation index (*F*_st_) and nucleotide diversity (θπ) were calculated using VCFtools (v0.1.16) with parameters--fst-window-size 40,000 and--fst-window-step 20,000. These calculations divided the genome into intervals of 40 KB with a 20 KB step. Candidate genomic regions were identified based on the following criteria: regions with extreme θπ ratios (5% left-tailed or 95% right-tailed) and significantly high *F*_st_ values (top 5%). Overlapping areas of low θπ ratios and high *F*_st_ values were identified as selective regions for HT, while overlaps of high θπ ratios and high *F*_st_ values were identified for CM. Genes within these candidate regions were designated as candidate genes (CGs).

### 2.5 RNA extraction and library construction

Using a circular skin sampler with a radius of 0.44 mm, collect skin sample from the posterior edge of the left foreleg scapula and store it in a cryovial containing RNA protective solution. Total RNA was extracted from 20 skin samples (10 from HT and 10 from CM groups) using TRIzol reagent (Invitrogen, CA, United States). The quality of the RNA samples was assessed, with results showing OD260/280 ratios between 1.8 and 2.0, OD260/230 ratios above 2.0, and RIN values ranging from 7.0 to 8.5 for all samples, indicating they were suitable for further experiments.

Strand-specific libraries were constructed, with ribosomal RNA removed to enhance circRNA library preparation by eliminating linear RNA. The library was initially quantified using Qubit and diluted to 1 ng/μL. The insert size was checked using the Agilent 2100 Bioanalyzer, showing a size range of 250–300 bp, as expected. Once the insert size was confirmed, the effective concentration of the library was accurately measured by qPCR. The concentration exceeded 2 nM, ensuring the library met quality standards. The twenty libraries were pooled and sequenced on the Illumina platform at Novogene Bioinformatics Technology Co., Ltd.

### 2.6 RNA sequencing quality control and analysis

FastQC was used to assess the quality of the raw RNA sequencing reads. The data were then processed to remove impurities and obtain clean reads for further analysis. Clean data were aligned to the sheep reference genome (ARS-UI_Ramb_v2.0) using HISAT2 (Kim et al., 2019). The transcripts were assembled and quantified with StringTie (Pertea et al., 2015), and the expression level of genes or transcripts was measured by fragments per kilobase million (FPKM). Differentially expressed genes (DEGs) were identified using edgeR (Robinson et al., 2010). Genes were considered differentially expressed if they met the criteria of |log2(fold change)| > 0 and p < 0.05.

### 2.7 Identification of key functional genes and pathway enrichment

The intersections of candidate genes (CGs) and differentially expressed genes (DEGs) were taken to identify key functional genes (KFGs). These genes were then analyzed for pathway enrichment using KEGG analysis on Kobas 3.0 (Bu et al., 2021), with Ovis_aries selected as the background organism. The hypergeometric test/Fisher’s exact test was used for statistical analysis, and pathways with a p-value < 0.05 were considered significantly enriched. Plots were generated using the R package ggplot2 (Villanueva and Chen, 2019).

Additionally, DEGs were submitted to the STRING database (Szklarczyk et al., 2023) to construct a protein-protein interaction network. The interaction network was visually edited using Cytoscape software (Smoot et al., 2011).

### 2.8 Machine-learning screening for signature genes

Neural Network (NNET) algorithm is a computational model inspired by biological neural systems and is widely used in tasks such as pattern recognition, classification, and regression. This study employed a Multilayer Perceptron (MLP) neural network for biomarker discovery and classification modeling, utilizing gene expression level as predictors to identify characteristic genes distinguishing different populations (HT vs. CM). The analytical framework comprised four key phases: data preprocessing, feature selection, model training, and validation. All implementations were conducted in R (v4.2.2) using the caret (v6.0-94) Kuhn, 2008 and nnet (v7.3-19) packages.

#### 2.8.1 Data preprocessing

The raw gene expression matrix was transposed to a sample × feature (gene) structure. An automated quantile-based criterion determined logarithmic transformation requirements: log2(x+1) transformation was applied to stabilize variance when either the 99th percentile exceeded 100 or the interquartile range surpassed 50 with a 25th percentile >0. In this study, the dynamic range of expression values spanned [QX1, QX6] (QX denotes specific quantile values), necessitating log2 transformation.

#### 2.8.2 Dataset partitioning

Stratified sampling allocated 70% of samples to the training set and 30% to the independent test set, preserving class proportions (Case/Control = 1:1) through the createDataPartition function from the caret package. A fixed random seed (74,521) ensured reproducibility.

#### 2.8.3 Feature selection

A two-stage feature screening strategy was implemented:

(1) Initial Screening: Boruta algorithm (100 iterations) evaluated global feature importance through statistical significance testing (p < 0.01) using shadow features.
(2) Refined Selection: A 10 × 10 repeated cross-validated neural network model was built on preselected features. Top 30 signature genes were identified via Variable Importance Measure (VIM) rankings computed through the connection weights algorithm, quantifying cumulative contributions of features to output nodes.

#### 2.8.4 Model construction and validation

(1) Hyperparameter Optimization: Grid search tuned hidden layer neurons (size∈{5,10}) and weight decay coefficients (decay∈{0.01,0.1}), optimized by the receiver operating characteristic area under the curve (AUC).
(2) Training Protocol: Parallel computing acceleration (doParallel package(v1.0.17)) enabled 5 × 20 repeated cross-validation: The training set was randomly partitioned into 10 folds, with 20 independent validation cycles to mitigate sampling bias.
(3) Performance Evaluation: Independent test set metrics included: Accuracy, AUC, Sensitivity and specificity.

#### 2.8.5 Statistical analysis

All visualizations (feature importance plots, ROC curves) were generated using ggplot2 (v3.4.2). Significance testing employed DeLong’s algorithm for ROC curve comparisons, with p < 0.05 considered statistically significant.

## 3 Results

### 3.1 Summary of genome and transcriptome sequencing

A total of 1,223.6 GB of clean data were obtained after genome resequencing, with an average of 24.97 GB per sample. The mapping rate across all samples ranged from 98.72% to 99.76%, and the average genome coverage depth (excluding N regions) ranged from 8.69X to 12.12X. Coverage of at least one base was above 97.67%, and coverage of at least four bases exceeded 91.54%. After mapping to the reference genome, SNP annotation was performed using ANNOVAR software, identifying 25,515,212 SNPs. The SNPs were located in intergenic (46.68%), intronic (40.74%), and exonic (1.59%) regions. The transition-to-transversion ratio (ts/tv) was 2.685, with 1816 exonic SNPs leading to stop-gain variations, 372 to stop-loss variations, and 154,692 to non-synonymous mutations.

For RNA-seq analysis, over 2.036 billion reads were analyzed, with 996 million from the HT group and 1.039 billion from the CM group. Each sample contained over 100 million reads, ranging from 84 million to 107 million. After trimming, approximately 29.4 million reads were removed, ensuring high data quality. Of the 2.006 billion clean reads, 1.036 billion (∼92.5%) were aligned to the genome, with 84.4% uniquely mapped and 8.1% mapped to multiple locations. The alignment rates for individual samples ranged from 89.2% to 94.7%. A summary of the RNA-seq datasets and mapping results is provided in Supplementary Table 4.

### 3.2 Screening of key functional genes

**FIGURE 2:**
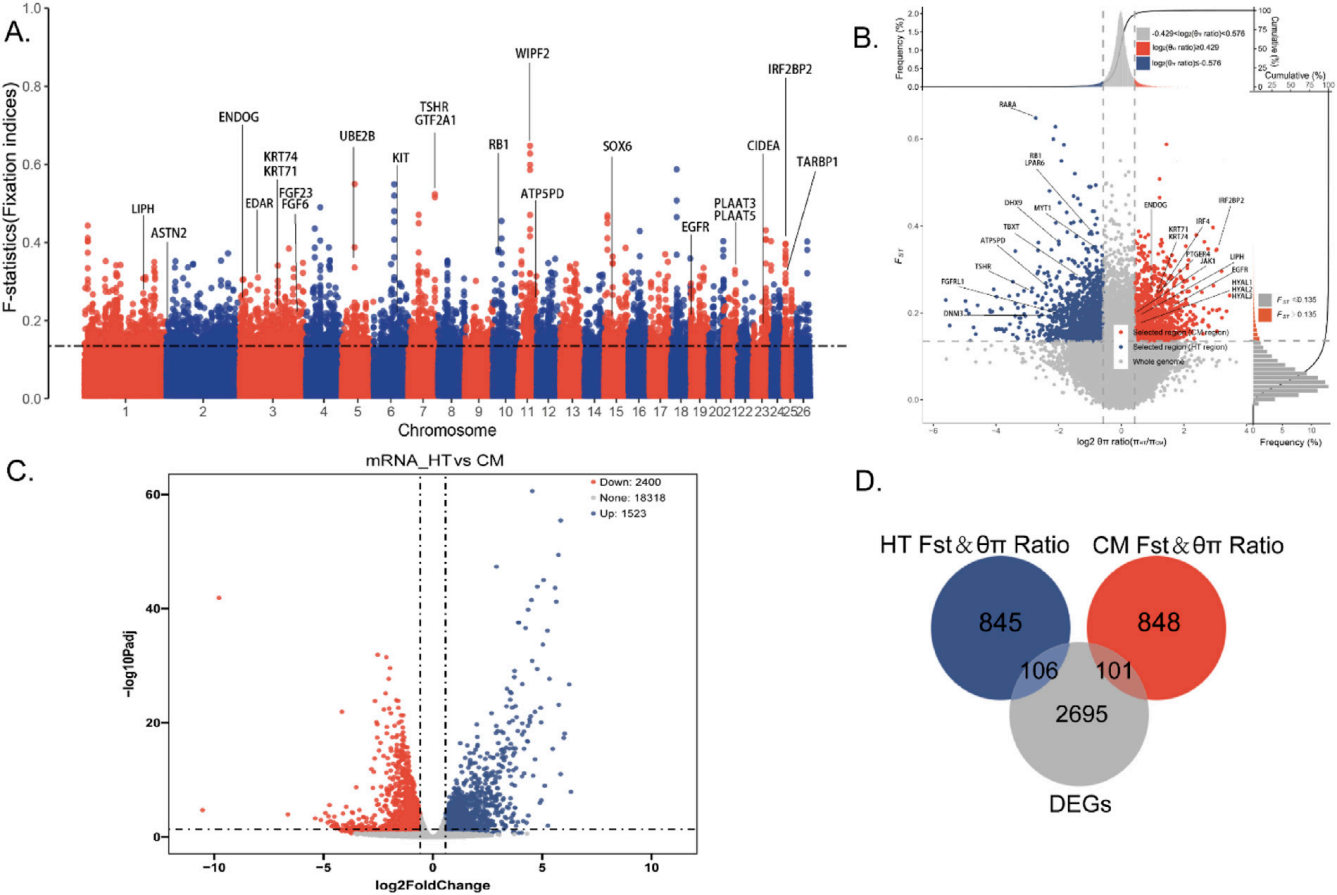
**(A)** *F*st analysis of Manhattan (Fixation Indices: 0–0.05: genetic differentiation between populations is small and negligible. 0.05–0.15: Moderate genetic differentiation between populations. 0.15–0.25: Genetic differentiation between populations. >0.25: There is significant genetic differentiation between populations). **(B)** *F*st and θπ ratio strategy selection signature analysis results are displayed (Note: The abscissa is the Log2π ratio, the ordinate is the *F*_st_ score, which corresponds to the frequency distribution map above and the frequency distribution map on the right, respectively, and the dot plot in the middle represents the corresponding *F*_st_ and Log2π ratio in different windows. The blue and redareas are the top 5% areas selected by *F*_st_ and Log2Pi ratio, blue represents Hetian sheep, and red represents Chinese merino sheep). **(C)** HT vs. CM mRNA gene level differential analysis results. **(D)** Venn diagram for *F*st and θπ ratio and differential expression analysis (red: CM *F*_st_ and θπ ratio; blue: HT *F*_st_ and θπ ratio; grey: DEGs).

The distribution of the genetic differentiation statistic *F*_st_ across the genomes of Hetian sheep (HT) and Chinese Merino sheep (CM) is summarized in Figure 2A and Supplementary Table 5. SNP annotation (top 5% of *F*_st_ scores) using the ARS-UI_Ramb_v2.0 sheep genome identified a total of 3,533 genes. Notable overlaps were found between the *F*_st_ outliers and known QTLs associated with wool traits (e.g., *KRT71*, *KRT74*, *IRF2BP2*), reproductive traits (e.g., *ASTN2*, *TSHR*, *GTF2A1*), and production traits (e.g., *PSAP*, *CDH23*, *UBE2B*), among others (Supplementary Table 6).

The combined selective sweep analysis of nucleotide diversity (θπ) and *F*_st_ yielded interesting results (Figure 2B; Supplementary Tables 7 and 8). The *F*_st_ and θπ ratio strategy revealed 949 genes in HT (Figure 2B, blue points) and 951 genes in CM (Figure 2B, red points). This analysis enabled complete differentiation of genes under selection pressure in both the HT and CM groups during growth.

RNA-seq data from the skin transcriptomes of 10 HT (experimental group) and 10 CM (control group) were compared, revealing a total of 2,902 differentially expressed genes (DEGs). Of these, 1,065 genes were upregulated and 1,837 were downregulated (Figure 2C; Supplementary Table 9).

A Venn diagram (Figure 2D) based on candidate genes from the *F*_st_ and θπ ratio and differential expression analysis identified 106 KFGs associated with single-coat fleece formation and 101 KFGs associated with double-coat fleece formation (Supplementary Table 10).

### 3.3 GO and KEGG enrichment analysis of KFGs

KFGs identified in the sheep genome and transcriptome using *F*_st_ and θπ ratio and differential expression analysis were further enriched for Gene Ontology (GO) and KEGG pathways.

**FIGURE 3:**
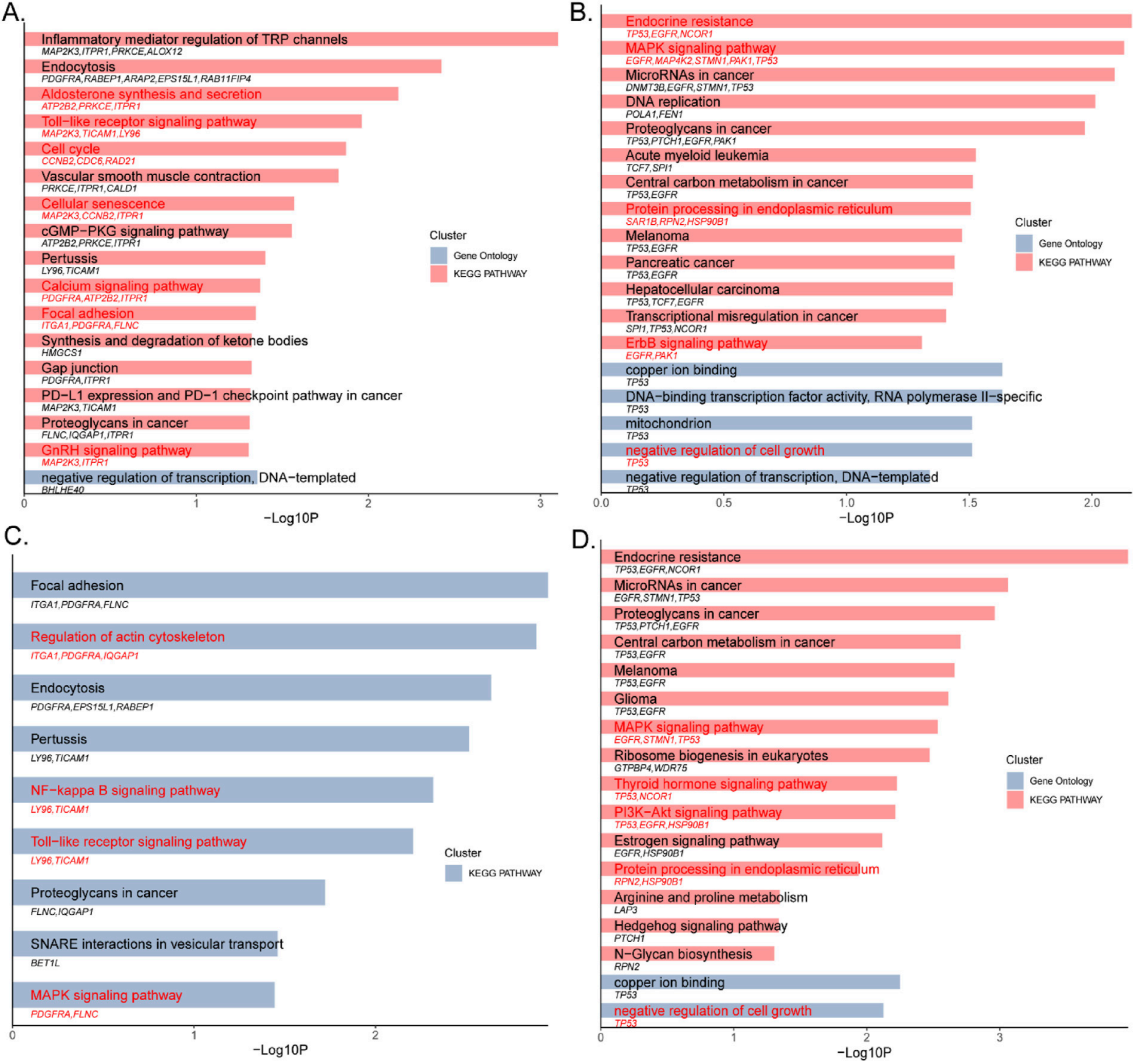
**(A)** The double-coated fleece trait (HT); **(B)** The single-coated fleece trait (CM); **(C)** The first interaction network of the double-coated fleece trait (HT); **(D)** The first interaction network of the single-coated fleece trait (CM).

For the double-coated fleece trait, 17 significant terms were identified (Figure 3A; Supplementary Table 11), including 16 KEGG pathways and 1 GO term (p < 0.05). Key pathways include Endocytosis (oas04144, *PDGFRA*, *RABEP1*, *ARAP2*, *EPS15L1*, *RAB11FIP4*), Toll-like receptor signaling (oas04620, *MAP2K3*, *TICAM1*, *LY96*), Cell cycle (oas04110, *CCNB2*, *CDC6*, *RAD21*), Cellular senescence (oas04218, *MAP2K3*, *CCNB2*, *ITPR1*), Pertussis (oas05133, *LY96*, *TICAM1*), Focal adhesion (oas04510, *ITGA1*, *PDGFRA*, *FLNC*), PD-L1 expression and PD-1 checkpoint pathway in cancer (oas05235, *MAP2K3*, *TICAM1*) and Proteoglycans in cancer (oas05205, *FLNC*, *IQGAP1*, *ITPR1*).

For the single-coated fleece trait, 31 significant terms were identified (Figure 3B; Supplementary Table 12), including 25 KEGG pathways and 6 GO terms (p < 0.05). These included Endocrine resistance (oas01522, *TP53*, *EGFR*, *NCOR1*), MAPK signaling pathway (oas04010, *EGFR*, *MAP4K2*, *STMN1*, *TP53*), protein processing in the endoplasmic reticulum (oas04141, *SAR1B*, *RPN2*, *HSP90B1*), DNA replication (oas03030, *POLA1*, *FEN1*), ErbB signaling pathway (oas04012, *EGFR*, *PAK1*) and negative regulation of cell growth (GO: 0030308, *TP53*).

### 3.4 Protein protein interaction network of DEGs

To further investigate the relationships between KFGs, the selected KFGs were imported into the STRING database to construct the protein-protein interaction (PPI) network, and Cytoscape was used to visualize the network. The cytoHubba plugin (Chin et al., 2014) was applied for network topology and node centrality analysis, allowing the identification of hub genes and subnetworks through network algorithms.

**FIGURE 4:**
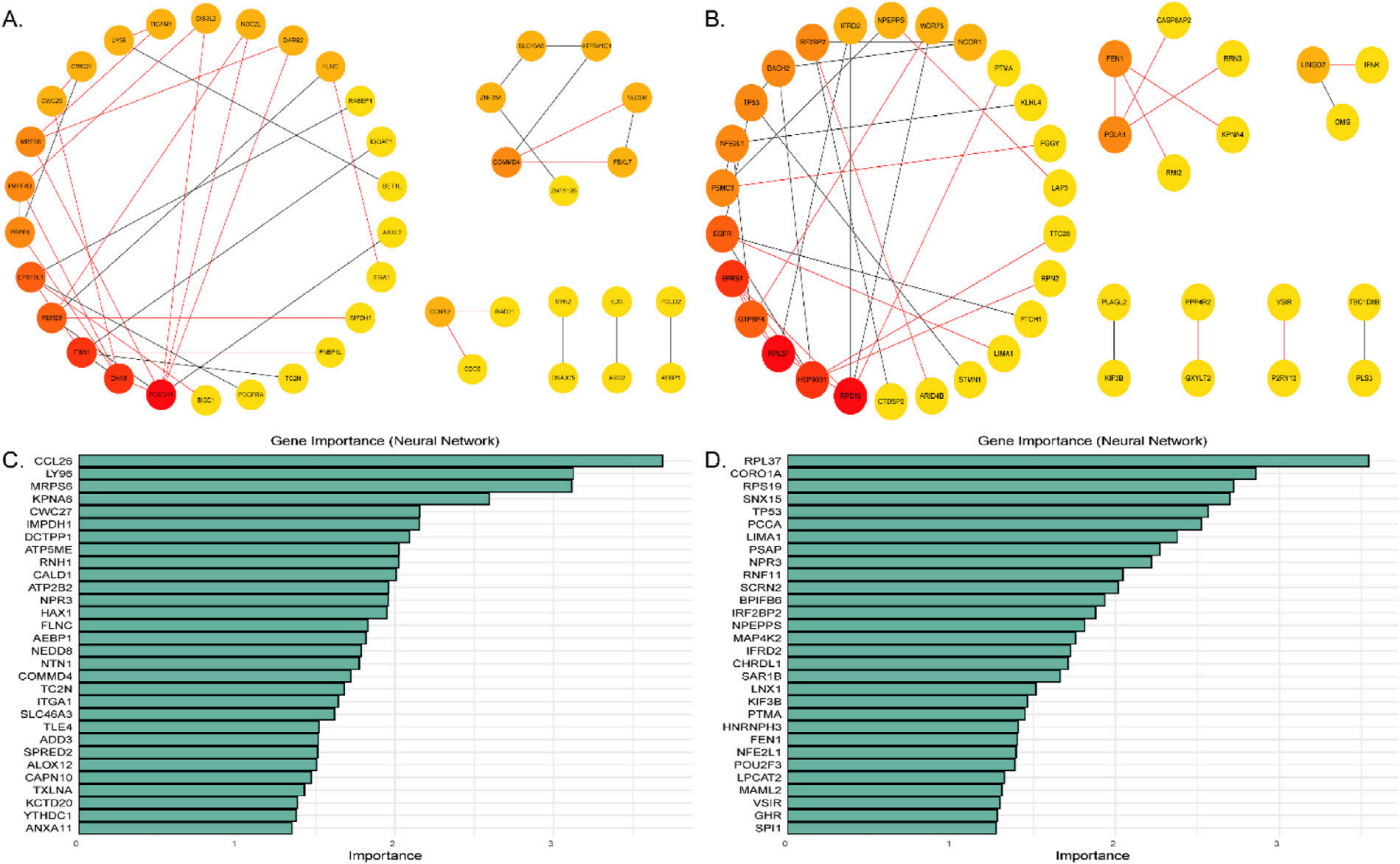
**(A)** Protein-protein interaction networks in the double-coated fleece trait. **(B)** Protein-protein interaction networks in the single-coated fleece trait. **(C)** Feature genes for double-coated fleece. **(D)** Feature genes for single-coated fleece. The abscissa coordinates show the importance of the features, sorted by the importance of the variables in descending order.

The analysis revealed that 42 KFGs formed six distinct PPI networks associated with the double-coated fleece trait (Figure 4A; Supplementary Table 13). Key genes such as *PDGFRA*, *RABEP1*, *EPS15L1*, *TICAM1*, *LY96*, *CCNB2*, *CDC6*, *RAD21*, *ITGA1*, and *FLNC* were present in these networks. In the first reciprocal network, the KFGs were primarily enriched in pathways such as regulatory actin cytoskeleton, NF-kappa B signaling, Toll-like receptor signaling, and MAPK signaling (Figure 3C; Supplementary Table 14). Among these genes, there was an interaction relationship between the *PDGFRA* and *EPS15L1*, *RABEP1* and *EPS15L1*, *CCNB2* and *CDC6*, *CCNB2* and *RAD21*, and *ITGA1* and *FLNC*.

A total of 43 differentially expressed genes formed seven protein-protein interaction (PPI) networks for the single-coated fleece trait (Figure 4B; Supplementary Table 15). Key genes such as *TP53*, *EGFR*, *NCOR1*, *STMN1*, *FEN1*, *PAK1*, *HSP90B1*, and *RPN2* were included in these networks. In the first reciprocal network, the KFGs were primarily enriched in pathways like MAPK signaling, thyroid hormone signaling, PI3K-Akt signaling, protein processing in the endoplasmic reticulum, and negative regulation of cell growth (Figure 3D; Supplementary Table 16). Among these genes, there was an interaction relationship between the *TP53* and *EGFR*, *EGFR* and *HSP90B1*, *EGFR* and *PTCH1*, *HSP90B1* and *RPN2*, and *NCOR1* and *IRF2BP2*.

### 3.5 Machine learning analysis of key functional gene

**FIGURE 5:**
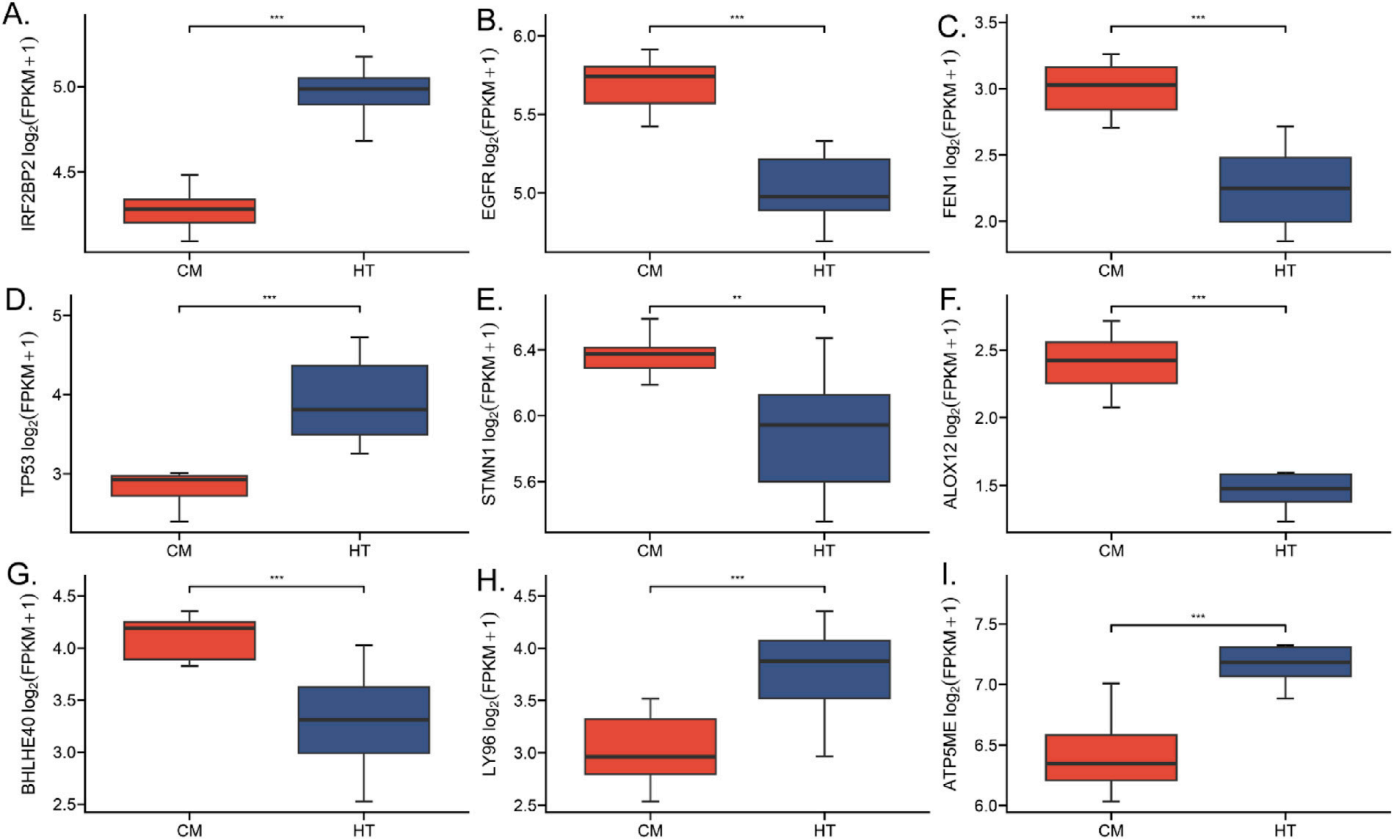
Expression levels of key genes associated with HT and CM coat types and wool growth traits. **(A)** IRF2BP2 gene. **(B)** EGFR gene. **(C)** FEN1 gene. **(D)** TP53 gene. **(E)** STMN1 gene. **(F)** ALOX12 gene. **(G)** BHLHE40 gene. **(H)** LY96 gene. **(I)** AT5ME gene.

Machine learning using the Neural Network (NNET) algorithm was applied to the 106 (single-coated fleece) and 101 (double-coated fleece) KFGs identified earlier, selecting the top 30 feature genes for each trait (Figures 4C, D). Among these, *IRF2BP2*, *TP53*, *FEN1*, *ALOX12*, *LY96*, *FLNC*, and *ATP5ME* were identified as highly important for wool growth traits and environmental adaptation. The results of transcriptome differential gene analysis also showed that the expression of these genes in HT and CM was extremely different (Figure 5). The performance of the constructed models was evaluated using tenfold cross-validation repeated ten times. Area Under the Curve (AUC) values were calculated based on Receiver Operating Characteristic (ROC) curves (Supplementary Figure S2). Detailed data used for the machine learning analysis is provided in Supplementary Tables 17 and 18.

## 4 Discussion

### 4.1 Enrichment analysis of KFGs in Chinese Merino and Hetian sheep

Enrichment analyses revealed that the KFGs enriched in Chinese Merino sheep were predominantly associated with pathways related to wool growth traits. In contrast, the KFGs enriched in Hetian sheep were linked not only to wool growth but also to adaptive traits. These differences reflect the distinct environments in which the two breeds have evolved. The Hetian sheep production region is characterized by a temperate continental desert climate with drought, high temperatures, and minimal rainfall. Such extreme climatic conditions, combined with long-term selective breeding, have resulted in the development of an exceptional local breed with remarkable drought and heat tolerance as well as high adaptability. On the other hand, the Chinese Merino sheep production region features a temperate continental climate with relatively mild ecological conditions. Decades of breeding efforts have focused on optimizing wool yield and fiber quality, particularly in terms of fineness, length, and crimp (Ciani et al., 2015; Wang et al., 2014).

In Chinese Merino sheep, 15 key functional genes (KFGs), including *TP53*, *FEN1*, *EGFR*, *TCF7*, *HSP90B1*, *PTCH1*, *NCOR1*, *MAP4K2*, *STMN1*, *PAK1*, *DNMT3B*, *POLA1*, *SAR1B*, *RPN2*, and *SPI1*, are involved in endocrine resistance, the MAPK signalling pathway, protein processing in the endoplasmic reticulum, the ErbB signalling pathway, and the negative regulation of cell growth, across 21 signalling pathways. These KFGs are likely to include potential regulators of wool growth in Chinese Merino sheep. Among these, the MAPK signalling pathway is well known for its close association with wool growth and development (Zhang et al., 2020; Öztürk et al., 2015; Sulayman et al., 2019; Lv et al., 2020). Endocrine resistance involves alterations in multiple signalling pathways and gene expression, with the ErbB pathway playing a key role in regulating cell proliferation and differentiation (Lu et al., 2021). Protein processing in the endoplasmic reticulum encompasses protein folding, modification, and transport (Potter and Nicchitta, 2002), processes that are critical for the proper formation of wool fibres (Yue et al., 2023). Additionally, the negative regulation of cell growth is essential for maintaining tissue homeostasis and preventing abnormal cell proliferation (Rousseau et al., 1996).

In Hetian sheep, 21 key functional genes (KFGs), including *MAP2K3*, *ALOX12*, *ARAP2*, *ATP2B2*, *BHLHE40*, *CALD1*, *CCNB2*, *CDC6*, *EPS15L1*, *FLNC*, *HMGCS1*, *IQGAP1*, *ITGA1*, *ITPR1*, *LY96*, *PDGFRA*, *PRKCE*, *RAB11FIP4*, *RABEP1*, *RAD21*, and *TICAM1*, are involved in aldosterone synthesis and secretion, the Toll-like receptor signalling pathway, the cell cycle, the calcium signalling pathway, focal adhesion, and the GnRH signalling pathway across 17 signalling pathways. These pathways are primarily associated with adaptation and wool growth in Hetian sheep. Aldosterone is an important corticosteroid that promotes sodium reabsorption and water retention, playing a critical role in the survival of Hetian sheep, which are adapted to saline soils (Bizzarri et al., 2016). The Toll-like receptor signalling pathway is vital to the immune system, with TLRs being central to inflammation, immune cell regulation, cell survival and proliferation, as well as adaptive immune responses by directing the differentiation of naïve T cells into effector T cells (Kulkarni et al., 2011; Kumar et al., 2024). The cell cycle pathway responds to external signals, such as growth factors, cytokines, and cell-to-cell contact, to regulate cell growth and division (Maddika et al., 2007). The GnRH signalling pathway is key in regulating the production and release of reproductive hormones, activating intracellular signalling cascades, and influencing both reproductive function and metabolic homeostasis (Kraus et al., 2001).

### 4.2 Genes associated with coat fleeced type in sheep

As early as 8,000 years ago, sheep were known to have a brown coat consisting of an outer layer of kemps (coarse hairs) that shed annually, along with a fine, woolly undercoat that also shed (Meadows et al., 2006). Over time, as food sources became more secure and sheep were domesticated, the characteristics of their wool evolved. It shifted from being coarse to fine, from short to long, and the coat fleece transitioned from double-layered to single-layered. In the present study, we identified two genes, *IRF2BP2* and *EGFR*, that are associated with coat fleece type.

*IRF2BP2* is involved in various cellular functions, including apoptosis, survival, and cell differentiation. It also plays a role in regulating the Hippo signaling pathway and acts as a tumor suppressor in hepatocellular carcinoma. Given the significant selection differences observed in the *IRF2BP2* gene in our study, we propose that *IRF2BP2* plays a pivotal role in the evolutionary transition from double-coated fleece to single-coated fleece. This is supported by previous studies, such as those by Demars et al. (2017); Lv et al. (2022); Sun et al. (2024), which identified mutations in *IRF2BP2* that are linked to wool traits, further suggesting its role in fleece composition.

*EGFR* (epidermal growth factor receptor) is a member of the HER family, known for its crucial role in skin and hair development. Research has demonstrated that subcutaneous injection of EGF into neonatal mice can delay the development of hair follicles and the epidermis. In adult sheep, EGF injection not only inhibits hair fiber production and stimulates mitosis in basal epidermal cells, but also induces hair follicle degeneration. Further studies in mice have shown that *EGFR* deficiency leads to abnormal expression of *LEF1*, which causes differentiation disorders in medullary cells (Mak and Chan, 2003; Amberg et al., 2019). In Hetian sheep, the EGFR-LEF1 pathway may regulate the alternating appearance of myelinated and unmyelinated regions within certain hair follicle units, contributing to the distinctive structure of heterotypical hair.

### 4.3 Genes related to wool growth and adaptation

The *TP53* gene encodes the p53 protein, a key regulator of cell division and cell death. It is involved in several signaling pathways directly related to wool growth, including the MAPK, Wnt, and PI3K-Akt pathways (Amberg et al., 2019; Chamcheu et al., 2019). Another critical gene, *FEN1*, encodes a flap endonuclease-1 enzyme that plays a central role in maintaining genome stability and replication. *FEN1* interacts with various proteins required for genome stability. In highly proliferative tissues such as bone marrow, testis, and thymus, *FEN1* expression is notably high. Studies of fusion gene expression in the epidermis (skin explants) of adult Fen1y/y mice revealed that proliferating keratin-forming cells were confined to the basal lamina and peri-follicular rondelles, suggesting that *FEN1* is crucial for hair follicle cell proliferation and hair growth (Kleppa et al., 2012). Stathmin 1 (*STMN1*) is a ubiquitously expressed cytosolic phosphoprotein involved in integrating diverse intracellular signaling pathways that control cell proliferation, differentiation, and activity. Depletion of Stmn1 leads to increased apoptotic death, accelerated degenerative transformation, and premature inhibition of hair follicle proliferation, highlighting its critical role in the hair follicle cycle (Yang et al., 2022; Zhang et al., 2021; Bichsel et al., 2016). Achidonate 12-lipoxygenase (*ALOX12*) is downregulated in senescent hair follicles. Its inhibition can prevent the breakdown and conversion of arachidonic acid in hair follicles, thereby promoting stratum corneum maturation (Zheng et al., 2020). *BHLHE40* Basic Helix-Loop-Helix Family Member E40 plays a pivotal role in cell differentiation. Knockdown of *BHLHE40* impairs the ability of epidermal cells to regenerate hair follicles (Wang et al., 2024). *LY96*, also known as *MD2*, is a small glycoprotein expressed by macrophages and dendritic cells. It is functionally related to Toll-like receptor 4 (*TLR4*) and plays a significant role in innate immunity by participating in the Toll-like receptor signaling pathway (Cao and Yang, 2024). *ATP5ME*, a subunit of ATP synthase, is involved in cellular energy metabolism and thermogenesis (Zhuang et al., 2023).

## 5 Conclusion

In summary, our research offers valuable insights into the regulatory mechanisms underlying wool growth, coat fleece type, and adaptability in wool growth observed in Hetian sheep. By comparing genetic differences between Hetian and Chinese Merino sheep, we identified both novel and previously reported candidate genes through selective sweep analysis, transcriptome differences, and machine learning approaches. In the Chinese Merino sheep population, we identified candidate genes associated with wool growth (*TP53*, *FEN1*, and *STMN1*) and coat fleece type (*IRF2BP2* and *EGFR*). In the Hetian sheep population, we identified key functional genes (KFGs) related to wool growth (*ALOX12*, *BHLHE40*, *RICTOR*, and *PIP4K2A*) and environmental adaptation (*LY96* and *ATP5ME*). Further research is required to fully elucidate the complex interactions between these KFGs and to develop effective strategies for their application in sheep breeding programs.

## Data Availability

The datasets presented in this study can be found in online repositories. The names of the repository/repositories and accession number(s) can be found in the article/Supplementary Material.
