# Supplementary material for: Machine learning driven multi-omics analysis of the genetic mechanisms behind the double-coat fleece formation in Hetian sheep
Source: Front Genet. 2025 Jun 11;16:1582244. doi: 10.3389/fgene.2025.1582244 (PMC12187771; doi:10.3389/fgene.2025.1582244)

Figure S1. The number of windows with less than 20 SNPs in the 10, 20, 30, 40, and 50KB window size


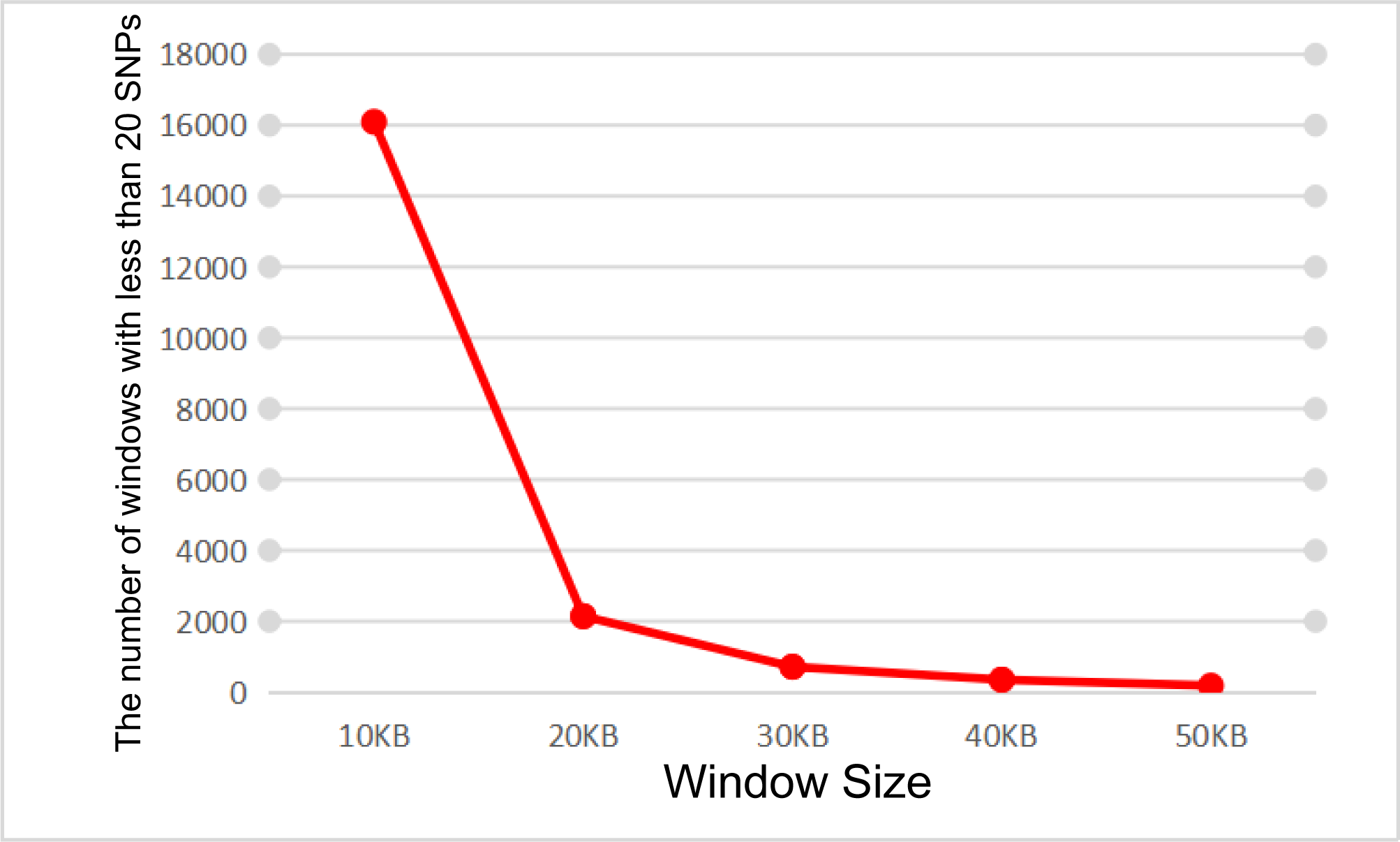


^1^The number of windows with less than 20 SNPs: By calculating the number of windows with less than 20 SNPs in different window sizes, we can find a window size that can not only improve the analysis efficiency but also enhance the interpretability of the results, so as to make the analysis results more practical.

Figure S2. SNP density plot


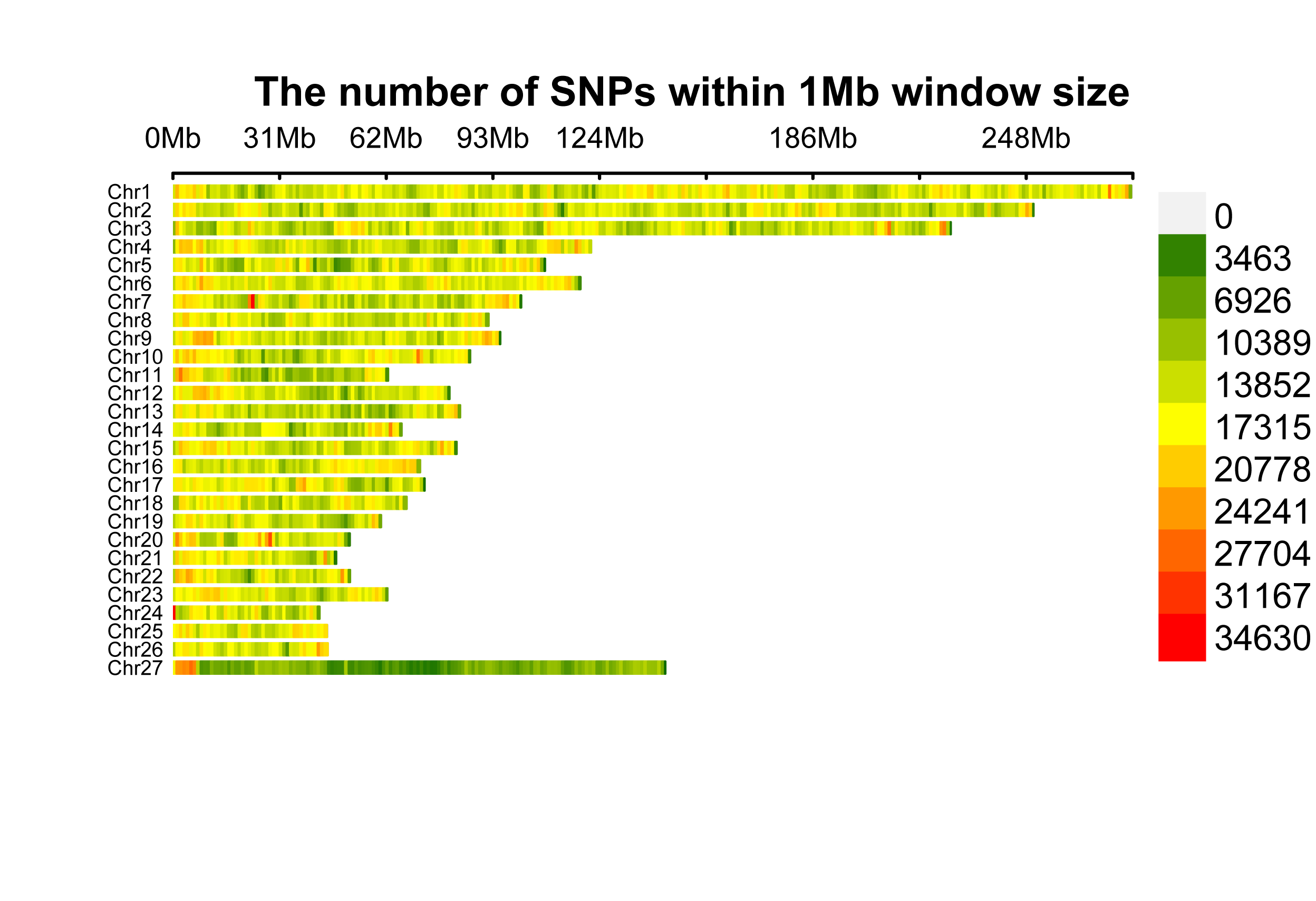


Figure S3. The AUC values were calculated based on the ROC curves. (A:HT;B:CM)


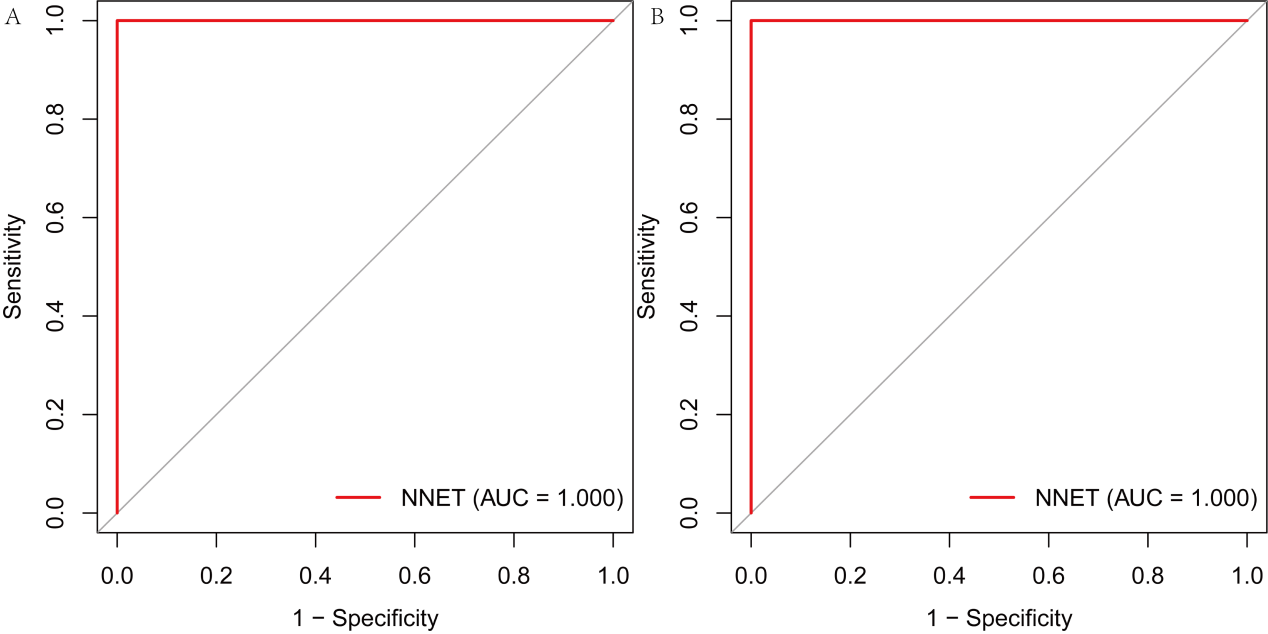

Supplement: Supplementary file 2 [file DataSheet1.docx]
